# Intraoperative endoluminal pyloromyotomy for reduction of delayed gastric emptying after pylorus preserving partial pancreaticoduodenectomy (PORRIDGE trial): study protocol for a randomised controlled trial

**DOI:** 10.1186/s13063-022-06032-2

**Published:** 2022-01-25

**Authors:** Matthias C. Schrempf, David R. M. Pinto, Sebastian Wolf, Bernd Geissler, Florian Sommer, Michael Hoffmann, Dmytro Vlasenko, Johanna Gutschon, Matthias Anthuber

**Affiliations:** Department of General, Visceral and Transplantation Surgery, University Hospital Augsburg, Augsburg, Germany

**Keywords:** Pylorus-preserving pancreaticoduodenectomy, Delayed gastric emptying, Pyloromyotomy, Randomised controlled trial

## Abstract

**Background:**

Pylorus-preserving pancreaticoduodenectomy (ppPD) is a standard surgical procedure for the treatment of resectable neoplasms of the periampullary region. One of the most common postoperative complications after ppPD is delayed gastric emptying (DGE) which reduces quality of life, prevents a timely return to a solid oral diet and prolongs the length of hospital stay. In a retrospective analysis, intraoperative endoluminal pyloromyotomy was associated with a reduced rate of DGE. The aim of this study is to investigate the effect of intraoperative endoluminal pyloromyotomy on postoperative DGE after ppPD in a randomised and controlled setting.

**Methods:**

This randomised trial features parallel group design with a 1:1 allocation ratio and a superiority hypothesis. Patients with a minimum age of 18 years and an indication for ppPD are eligible to participate in this study and will be randomised intraoperatively to receive either endoluminal pyloromyotomy or atraumatic stretching of the pylorus. The sample size calculation (*n*=64 per study arm) is based on retrospective data. The primary endpoint is the rate of DGE within 30 days. Secondary endpoints are quality of life, operation time, estimated blood loss, length of hospital stay, morbidity and mortality.

**Discussion:**

DGE after ppPD is a common complication with an incomplete understood aetiology. Prevention of DGE could improve outcomes and enhance quality of life after one of the most common procedures in pancreatic surgery. This trial will expand the existing evidence on intraoperative pyloromyotomy, and the results will provide additional data on a simple surgical technique that could reduce the incidence of postoperative DGE.

**Trial registration:**

German Clinical Trials RegisterDRKS00013503. Registered on 27 December 2017.

## Background

Partial pancreaticoduodenectomy (PD) is considered standard therapy for resectable malignant neoplasms of the pancreatic head, distal common bile duct and papilla of Vater as well as precursors with malignant potential, such as intraductal papillary mucinous neoplasia (IPMN). The classical partial pancreaticoduodenectomy with resection of the distal stomach was modified by Traverso in the 1970s by introducing the pylorus-preserving technique (pylorus-preserving partial pancreaticoduodenectomy ppPD) [1]. Both procedures have been shown to be equally effective in terms of morbidity, overall survival and tumour recurrence [2, 3].

One of the most common complications after both procedures is a delayed gastric emptying (DGE) affecting up to 61% of patients [4]. Although DGE is not a lethal complication, it is associated with reduced quality of life and prolonged length of hospital stay [5, 6]. Severe cases may even lead to delayed initiation of subsequent therapeutic measures such as adjuvant chemotherapy [6].

DGE after PD was first described by Warshaw and Torchiana in 1985, but prior to the introduction of the International Study Group of Pancreatic Surgery (ISGPS) definition of DGE in 2007, the term was inconsistently defined by different authors, which severely limited the comparability of studies on DGE until then [7, 8].

The underlying mechanisms of DGE are not yet fully elucidated, but spasm of the pyloric muscle, devascularisation of the pylorus and postoperative hormonal changes may contribute to the development of DGE [9–12]. Intra-abdominal complications such as pancreatic fistula, anastomotic leakage and formation of haematoma or abscess have been associated with the development of DGE in several studies [13–15].

Various modifications including route of reconstruction, pyloric dilatation, pyloric resection and their impact on DGE have been studied with conflicting results [4, 16–19]. Meta-analyses and larger randomised trials using the “International Study Group of Pancreatic Surgery” definition of DGE failed to demonstrate an association between modifications studied and DGE [4, 20–22].

Pyloromyotomy during ppPD was first investigated by Kim et al. who performed a Fredet-Ramstedt type pyloromyotomy and antroplasty in 47 consecutive patients undergoing ppPD [9]. The reported DGE incidence of 2.2% can be partially explained by the underlying strict individual definition of DGE consisting of the inability to tolerate any oral intake, including a liquid diet, for three consecutive days in the absence of any attributable complications. However, a comparison with patients from the same institution treated by the same authors before the introduction of Fredet-Ramstedt pyloromyotomy showed a reduction in DGE rates with pyloromyotomy. Furthermore, endoscopic approaches to gastroparesis support the idea of performing endoluminal pyloromyotomy to reduce DGE [23–25]. In gastroparesis with underlying increased pyloric tone and pylorospasm, peroral endoscopic endoluminal pyloromyotomy can permanently improve gastric emptying [26]. Assuming that pylorospasm and pyloric dysregulation play a role in the development of DGE, we introduced an intraoperative endoluminal pyloromyotomy for patients undergoing ppPD at our institution several years ago. A retrospective analysis of our technique showed that intraoperative pyloromyotomy was associated with a significant reduction in DGE in a multivariate analysis [27]. These promising results encouraged us to further investigate our findings in prospective and randomised trial.

### Objectives

This is a randomised single-centre trial of patients undergoing pancreaticoduodenectomy with a superiority hypothesis: intraoperative pyloromyotomy is associated with less DGE compared to multidimensional atraumatic stretching of the pylorus. The results of this trial may provide additional evidence for the discussion on surgical approaches to reduce DGE and could have an impact on a commonly performed operation in pancreatic surgery.

## Methods

### Trial design

**Fig. 1:**
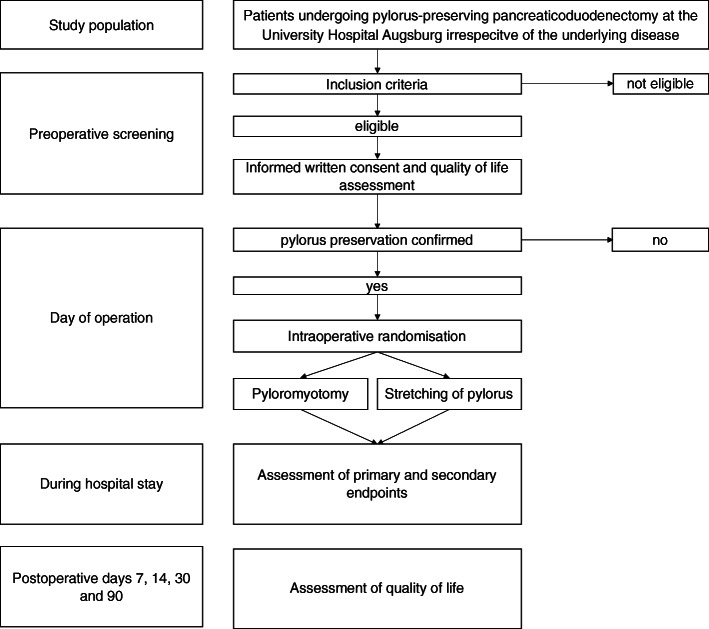
Trial flow chart

This randomised trial features parallel group design with a 1:1 allocation ratio and a superiority hypothesis (intraoperative endoluminal pyloromyotomy during ppPD is associated with a reduced DGE rate compared to multidimensional atraumatic stretching of the pylorus). The flowchart of the study is visualised in Fig. 1.

### Study setting and population

The study is conducted as a single-centre trial at the University Hospital Augsburg, Germany.

### Eligibility criteria

Patients with a minimum age of 18 years and an indication for a ppPD irrespective of the underlying disease who are scheduled for an elective surgery are eligible to participate in this study. All patients must provide written informed consent prior to participation. Patients who are unable to give consent, patients under legal guardianship, and patients participating in another intervention study that has a potential impact on the endpoint of this study will be excluded from participation.

### Informed consent and recruitment

All patients must provide written informed consent prior to participation. Informed consent for study participation and data collection will be obtained preoperatively by a surgeon at the University Hospital Augsburg. To ensure sufficient participant enrolment to achieve the sample size patients, scheduled for ppPD will be offered participation in the trial at the time they provide informed consent for surgery. Informed consent for study participation will be obtained during the explanation of the planned surgical procedure and the study intervention. Written information about the surgical procedure itself and the study intervention will be provided to support informed consent.

### Endpoints

#### Primary endpoint

The primary endpoint is defined as the rate of DGE according to the ISGPS definition within 30 days after ppPD. The two study groups (pyloromyotomy vs. no pyloromyotomy) are compared with respect to the primary endpoint.

#### Secondary endpoint

Secondary endpoints include the grade of DGE according to the ISGPS definition (proportion of with DGE Grade A, B, or C, see definition below), operative time (minutes), estimated blood loss (millilitres), overall morbidity (absolute number of complications and percentages in each group) according to the Clavien-Dindo classification [28] in-hospital mortality (absolute number of deaths and percentages in each group, cause of death), length of hospital stay (days from day of surgery to discharge), rate of primary DGE (defined as DGE in the absence of intraabdominal complications, absolute numbers, percentages) and postoperative quality of life. Serious adverse events will be documented, absolute and relative frequency, severity and relationship to the intervention will be reported and compared between intervention groups. Quality of life assessment in this study will be performed with the *European Organisation for Research and Treatment of Cancer* (EORTC) *Quality of Life Questionnaire Core 30* (QLQ-C30), a valid and widely used questionnaire in cancer patients [29], in combination with the supplementary module for patients with pancreatic cancer QLQ-PAN26. Quality of life data are assessed preoperatively and postoperatively on days 7, 14, 30, and 90. The assessment schedule is visualised in the SPIRIT figure (Table 1).

**Table 1:** Schedule of enrolment, intervention and assessment

Timepoint/period	Study period						
	Day before surgery	During surgery	During hospital stay	POD 7	POD 14	POD 30	POD 90
Eligibility screening	**X**						
Informed consent	**X**						
Randomisation and allocation		**X**					
Intervention (both arms)		**X**					
Assessment of quality of life	**X**			**X**	**X**	**X**	**X**
Assessment of primary and secondary endpoints		**X**	**X**				

*POD* postoperative day

### Definition of delayed gastric emptying

For this study, the ISGPS definition of DGE was applied [8]. Grade A DGE was present if the nasogastric tube (NGT) was still in place or reinserted between postoperative days (POD) 4 and 7, or if the patient was unable to tolerate a solid oral diet by POD 7. Patients were found to have grade B DGE if the NGT was still in place or reinserted between POD 8 and POD 14, or if the patient could not tolerate a solid oral diet by POD 14. If the NGT was still in place or reinserted after POD 14, or if the patient could not tolerate a solid diet by POD 21, the DGE was classified as grade C in accordance with the ISGPS definition.

### Surgical procedures

**Fig. 2:**
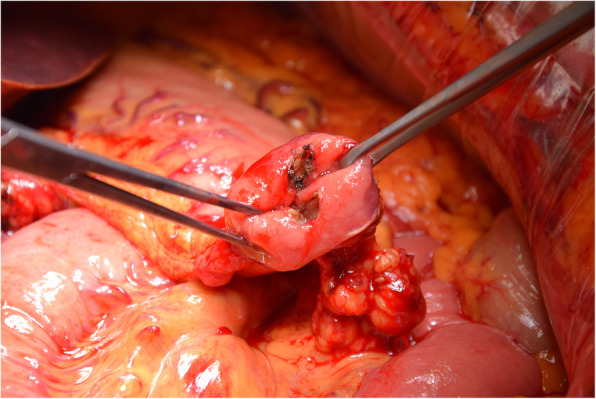
Intraoperative endoluminal pyloromyotomy

Participating patients will be randomised intraoperatively after confirmation of absence of macroscopically visible metastases and technical resectability with pylorus preservation. The duodenum is transected 2 to 4 cm distal to the pylorus with a linear stapling device. Pancreaticojejunostomy and hepatojejunostomy are performed with end-to-side anastomoses. One of two different surgical manoeuvres is performed prior to the creation of the duodenojejunostomy, depending on the results of randomisation. In patients randomised to the “pyloromyotomy group”, an endoluminal pyloromyotomy using electrocautery is performed to transect the mucosa, submucosa and circular pyloric muscle anteriorly and posteriorly at the 12 and 6 o’clock positions (Fig. 2). In patients randomised to the “no pyloromyotomy group”, a Gross-Maier dressing forceps is used to atraumatically stretch the pyloric muscle before creating the duodenojejunostomy. The duodenostomy is performed by creating an end-to-side anastomosis with a single-layer running monofilament atraumatic suture technique. In all patients, reconstruction is performed with an omega loop in an antecolic fashion and a side-to-side Braun jejunostomy approximately 15 cm distal to the duodenojejunostomy. A nasogastric tube is placed in the stomach during the operation. The nasogastric tube is removed on the first postoperative day unless medical reasons prevent the removal of the nasogastric tube. The procedures were performed by or under the direct supervision of board-certified pancreatic surgeons who meet criteria for pancreatic competence centres set by the German Society for General and Visceral Surgery.

### Methods against bias

#### Randomisation

Randomisation will minimise selection bias and potential confounding. Participants will be randomised intraoperatively after absence of macroscopically visible metastases and technical resectability with pylorus preservation has been confirmed. If premature randomisation has occurred and neither pyloromyotomy of stretching of the pylorus can be performed, randomisation will be discarded, the participant will be excluded from the study and no further data will be collected. If the intraoperative findings result in a total pancreatectomy, the patients will be excluded from the study and the randomisation, if already performed, will be discarded, the participant will be excluded from the study and no further data will be collected. Allocation to one of the two study arms will be carried out using a web-based randomisation tool which generates a random allocation sequence (https://www.randomizer.at). Block randomisation will be performed to ensure equal-sized groups. The size of the individual blocks will only be disclosed after the study has been completed so as not to allow prediction of group allocation. A sufficient number of subjects will be recruited according to the sample size calculation to prevent random errors and to ensure sufficient power to test the hypothesis of the primary endpoint. Randomisation will be performed by individuals not involved in the surgical procedure, data evaluation, data analysis, postoperative care, and follow-up of the patients.

#### Blinding

Patients as well as individuals involved in data collection, endpoint assessment and quality of life assessment will be blinded to group allocation to reduce information and measurement bias. As the study intervention is performed as part of a surgery under general anaesthesia and the study intervention is neither visible nor disclosed to the patient, patients can be considered blinded. Participating surgeons will be instructed which treatment procedures are applicable in each group. Blinding of surgeons is not feasible due to the nature of the interventions. Unblinding is possible in case of urgent medical reasons. If the treating surgeon and one of the investigators jointly decide that unblinding is necessary, unblinding can be done by any person authorised to randomise.

#### Intraoperative and postoperative management

Standardised instruction of participating surgeons in the application of the trial interventions reduce performance bias. All patients receive 100 μg of somatostatin subcutaneously intraoperatively at the time creation of the pancreaticojejunostomy and 100 μg of somatostatin subcutaneously three times daily thereafter until postoperative day 5, based on the internal standard.

### Sample size calculation

The sample size calculation is based on the primary endpoint "rate of delayed gastric emptying". A retrospective analysis from all patients who underwent ppPD at our institution between 2015 and 2017 revealed a DGE rate of 40.9% in patients who received intraoperative pyloromyotomy compared to a DGE rate of 66.7% in patients who did not receive pyloromyotomy, a difference in absolute risk of 25.8%. The calculation was carried out according to the method described by Campbell et al. for two-group assessments with binary outcomes [30]. Based on these figures, a sample size of 58 patients per treatment group is required to ensure a power of 80% at a two-sided significance level of 5%. To compensate for potential dropouts that would lead to dilution of the treatment effect, an additional 10% of patients will be recruited and randomised. Thus, 64 patients per group will be enrolled, resulting in a total number of 128 patients.

### Data collection

All data will be documented in hard copy case report forms. Quality of life data are recorded using the EORTC QLQ-C30 form in combination with the supplementary module for pancreatic cancer QLQ-PAN26. Both forms can be obtained from the *European Organisation for Research and Treatment of Cancer* (www.eortc.org). The completed CRFs will be reviewed by one of the investigators or an authorised sub-investigator. All data collected according to the study protocol will be manually transferred from the case report forms to an electronic SPSS file (IBM, Armonk NY, USA). Regular reviews of the correct data transfer are conducted by assessors at the study site. The electronic data will be stored in a protected folder on a server at University Hospital Augsburg. Paper-based data are stored in a locked office at the study site. In order to improve participant retention and collection of follow-up data, participants will be reminded regularly to complete the quality-of-life questionnaires.

### Pseudonymisation

Data is assessed and analysed in pseudonymised form. For this purpose, a randomly generated numerical four-digit code is assigned to each participant. Access to the original data and the pseudonymisation lists is restricted to the staff of the Department of General-, Visceral and Transplant Surgery at the University Hospital Augsburg. All identifying data will be deleted as soon as they are no longer used for research. The trial coordinators, the investigators and independent statisticians of the trial will have access to the final dataset.

### Data monitoring, data management and patient safety

The trial intervention is a low harm intervention that has been routinely performed at our institution for several years before initiation of the trial. A retrospective analysis of the study intervention which has been published did not reveal any safety concerns [27]. Therefore, there is no anticipated harm and no compensation for trial participation. Any complications will be reported to the Ethics Committee as part of the final report and included in the publication of the study results. Even without trial participation, patients who undergo pylorus-preserving pancreaticoduodenectomy at our institution will receive either pyloromyotomy or stretching of the pylorus as part of the routine surgery depending on the preference of the surgeon in charge.

To improve data quality, several measures were implemented. An internal data monitoring during the study is carried out by individuals not involved in data collection, patient recruitment or randomisation, but who have sufficient knowledge in conducting clinical trials. Annual reports on the participation rate and the status of the follow-up will be prepared. Data monitoring will be discussed with the investigators to review the progress of the study address potential problems. As part of the data monitoring process and to ensure proper data transfer from raw data and paper case report forms to data sets, all collected study data are entered into two electronic data sets by different persons. These two parallel data sets are compared at the end of the study using a computerised process. Any discrepancies between the two data sets are manually checked and corrected. An independent close-out audit is conducted before the data analysis, after the follow-up for the last participant has been completed.

### Data analysis plan

Continuous data will be presented as mean ± standard deviation or median with interquartile range, depending on the distribution. Categorical data will be presented as numbers with percentages. Approximately normally distributed continuous variables will be compared using the independent *t*-test. Non-normally distributed continuous variables will be compared using the Mann-Whitney-*U* test. Categorical data including the primary endpoint will be compared using the *χ*^2^ test. Fisher’s exact test will be used for categorical data if the requirements for *χ*^2^ test are not met. A two-sided *P* < 0.05 is considered significant. The primary analysis will be conducted in an intention-to-treat (“as randomised”) population. Discarded randomisations as defined in the randomisation section will be excluded from analysis. A multivariate analyisis of the primary endpoint will be performed which will include risk factors with a potential association with DGE (*P* < 0.15). Quality of life data will be analysed in accordance with the EORTC QLQ-C30 scoring manual [31]. An interim analysis was omitted because a sample size calculation was performed, and the study intervention regularly performed in ppPD at our institution is considered a low harm intervention that was not associated with an increased complication rate in a retrospective analysis. A subgroup analysis is performed for patients without intra-abdominal complications to assess the rate of DGE in this patient population.

Cases with missing data for the primary endpoint data resulting from the inability to assess the primary endpoint will be excluded from the primary endpoint analysis. Possible reasons for not being able to assess the primary endpoint are death of a participant before POD 7, unless the patient was able to tolerate a solid diet at that time (see ISGPS definition of DGE above), and the occurrence of a lymphatic fistula with the need for total parenteral nutrition.

Missing quality of life data will be addressed according to the recommendations of the EORTC scoring manual for quality-of-life questionnaires.

## Discussion

DGE is a common complication after ppPD. Despite numerous efforts to reduce DGE, most surgical modifications have not been associated with improved rates of DGE in prospective randomised controlled trials or meta-analysis. This underscores the difficulty of preventing and treating DGE. Several factors a likely to contribute to the development of DGE. Intraoperative endoluminal pyloromyotomy could reduce the potential impact of pyloric dysregulation or pyloric spasm on the development of DGE after ppPD. To our knowledge, this is the first randomised trial to investigate the impact of intraoperative pyloromyotomy on DGE. Results from a meta-analysis and larger randomised trials investigating the influence of pyloric resection during ppPD question pyloric dysregulation as the main aetiology for DGE [20, 22, 32]. Instead, these findings support a multifactorial aetiology and highlight the incompletely understood mechanisms leading to DGE. In our retrospective analysis, we showed that intraoperative endoluminal pyloromyotomy was associated with a significant reduction in the rate of DGE in both univariate and multivariate analyses [27]. Because retrospective studies are inherently subject to certain limitations such as incomplete documentation, interpretation bias and selection bias, we decided to verify our results with a prospective randomised trial.

As quality of life is essential for every cancer patient, it has understandably become an increasing focus of cancer research in recent years [33]. Therefore, we decided to include quality of life as a secondary endpoint, which will be measured with an established and validated questionnaire. This allows us to additionally analyse a strongly patient-centred endpoint.

The trial is currently recruiting patients and the results will provide additional data on a simple surgical technique that could reduce the incidence of postoperative DGE.

## Trial status

The trial has been registered in the German Clinical Trials Register (DRKS00013503), on 27 December 2017. The full WHO trial registration dataset is available through the WHO ICTRP search portal. The protocol version is 2017-03; 16 October 2017, Recruitment began on 16 February 2018. The expected date for recruitment completion is September 2022.

## Data Availability

After publication of the study results, a fully anonymised data set and the statistical code can be made available upon justified scientific request and after ethical approval has been granted. Depending on the extent of the data use and the planned research, either appropriate credit or co-authorship must be granted to the authors of this study. A sample of the CRF used for this study can be provided upon justified scientific request.

## Abbreviations

DGE: Delayed gastric emptying
EORTC: *European Organisation for Research and Treatment of Cancer*
IPMN: Intraductal papillary mucinous neoplasia
ISGPS: *International Study Group of Pancreatic Surgery*
NGT: Nasogastric tube
POD: Postoperative day
ppPD: Pylorus-preserving pancreaticoduodenectomy
